# Crown Procyanidin Tetramer: A Procyanidin with an Unusual Cyclic Skeleton with a Potent Protective Effect against Amyloid-β-Induced Toxicity

**DOI:** 10.3390/molecules24101915

**Published:** 2019-05-18

**Authors:** Liming Zeng, Pere Pons-Mercadé, Tristan Richard, Stéphanie Krisa, Pierre-Louis Teissèdre, Michael Jourdes

**Affiliations:** 1 Univ. Bordeaux, ISVV, EA 4577, Œnologie, 210 Chemin de Leysotte, F-33140 Villenave d’Ornon, France; liming.zeng@changins.ch (L.Z.); pepome@gmail.com (P.P.-M.); tristan.richard@u-bordeaux.fr (T.R.); Stephanie.Krisa@u-bordeaux.fr (S.K.); pierre-louis.teissedre@u-bordeaux.fr (P.-L.T.); 2 INRA, ISVV, USC 1366 INRA, IPB, 210 Chemin de Leysotte, F-33140 Villenave d’Ornon, France

**Keywords:** crown procyanidin, procyanidin, condensed tannin, grape, red wine

## Abstract

The structure of a new procyanidin tetramer, which we call a crown procyanidin tetramer, with an unprecedented macrocyclic structure has been characterized for the first time. Its comprehensive spectroscopic analysis revealed that it is a symmetric procyanidin tetramer composed of four (−)-epicatechin sub-units linked alternatively via 4β→8 or 4β→6 B-type interflavanyl linkages to form the macrocyclic structure. This NMR-characterized carbon skeleton has never been reported before for procyanidins in grape or in wine, neither in the plant kingdom. Surprisingly, the crown procyanidin tetramer appeared to be specifically localized in grape skin, contrasting with the oligomeric and polymeric procyanidins present in seed, skin, and bunch stem. Moreover, this crown procyanidin tetramer showed promising protective effects against amyloid-β induced toxicity.

## 1. Introduction

Polyphenols are widely distributed in plant-derived foods and beverages such as grapes, red wine, nuts, tea, olive oil, spices, apples and chocolate, in which they contribute to multiple sensorial properties such as flavor, color, and taste (e.g., astringency and bitterness) [1]. In the Western diet, the regular intake of fruits and vegetables with high polyphenolic content is strongly recommended, principally because polyphenolic compounds can play important roles on human health by mainly decreasing the risk of degenerative diseases [2]. Besides, the potential health benefits of condensed tannins, such as antioxidant effects [3], anti-inflammatory effects [4], cardiovascular system amelioration effects [5], and hyper tension diminution effect [6] have been proved in several studies [7]. These natural products are also the main active compounds in numerous plant extracts used in traditional Eastern medicines [8,9]. Phenolic compounds also play an important role in plant growth and reproduction, providing protection against biotic and abiotic stress, such as pathogen and insect attack and UV radiation [10,11].

Proanthocyanidins, also known as condensed tannins represent the most abundant class of natural phenolic compounds after lignin in the plant kingdom e.g., grapes [12,13]. In grape proanthocyanidins derived from the polymerization of five monomeric units: (+)-catechin, (−)-epicatechin, (−)-epigallocatechin, epicatechin gallate, and epigallocatechin gallate. They can be found with different degrees of polymerization, with as much as 80 flavan-3-ols units linked together. According to their localization in the berries, their degrees of polymerization ranged between 2 and 16 in grape seeds, while it can be from 2 to 83 in grape skin [14]. In the oligomeric and polymeric structures, the flavan-3-ols units are link together by B-type interflavanoid linkages between the carbon C4 of the upper units and the carbon C8 or C6 of the bottom unit [2].

Herein, we report for the first time the isolation and structural elucidation of a new procyanidin tetramer with an unusual macrocyclic carbon skeleton that has never been characterized before in the plant kingdom and we have called a “crown procyanidin tetramer.” Together with its structural characterization (i.e., mass spectrometry, NMR and acidic depolymerization) and localization in grape berries (i.e., seed, skim, or bunch stem), its neuroprotective activity against amyloid-β peptide (Aβ)-induced neurotoxicity in cultured PC12 cells using the MTT assay was also evaluated.

## 2. Results

Following our previous investigation of grape proanthocyanidin, one surprisingly polar condensed tannin tetramer exhibiting lower retention compared to usual oligomeric condensed tannins was detected by liquid chromatography coupled with high resolution mass spectrometry in grape extract and in red wine. According to its fragmentation pattern obtained by high resolution mass spectrometry, this new compound belongs to the procyanidin family. In order to obtain a full structural characterization, this tetramer was isolated using a three-step purification strategy in order to obtain the pure crown procyanidin tetramer (**1**) from red wine.

Tetramer **1** was obtained as a white powder, and its molecular formula was determined as C_60_H_48_O_24_ from the HRESIMS ion at *m*/*z* 1153.2597 [M + H]^+^ (calcd. 1153.2608). The observed fragment ions at 865.1963, 577.1334, and 289.0700 corresponded to the loss of one, two, and three flavan-3-ols units with a loss of 288.0634 Da (-C_15_H_12_O_6_) per unit. Multiple characteristic fragments of the flavan-3-ol skeleton with a loss of 152.0473 Da (-C_8_H_8_O_3_), and 126.0317 Da (-C_6_H_6_O_3_) were observed from the pseudo-molecular ion at 1153.2597 resulting from a Retro-Diels-Alder fission (RDA) and heterocyclic ring fission (HRF), respectively (Figure S1) [15]. Thus, **1** appeared to be a tetrameric compound composed of four flavan-3-ol monomers such as catechin and epicatechin. To start the structural identification of **1**, an aliquot was submitted to an acidic depolymerization procedure in the presence of an excess of phloroglucinol [16]. This chemical depolymerization strategy cleaved the B type (i.e., C4-C8 and C4-C6) interflavanyl linkages and then released the upper units of the condensed tannin as phloroglucinol adducts on the flavan-3-ol unit while the terminal units were simply released as flavan-3-ol unity. The obtained reaction mixture from the phloroglucinolysis of **1** was analyzed by UPLC-Q-Tof and showed the release of an (−)-epicatechin phloroglucinol adduct as well as some oligomeric phloroglucinol adducts, namely two dimer phloroglucinol adducts and two trimer phloroglucinol adducts (Table 1).

However, no monomeric flavan-3-ol units without addition of phloroglucinol were detected. This surprising result indicates the absence of a terminal unit in the isolated oligomeric condensed tannins. Indeed, if a terminal unit was present in the structure of **1**, flavan-3-ol should have been released during this chemical depolymerization. Moreover phloroglucinolysis of **1** was also performed over a longer period (45 min) in order to reach a complete depolymerization. In these more drastic conditions, only the (−)-epicatechin phloroglucinol adduct was released, and still no terminal unit (i.e., flavan-3-ol monomers released without the addition of phloroglucinol) could be detected. These results indicate that **1** could be a tetramer composed of four (−)-epicatechin units with one terminal unit.

**Figure 1:**
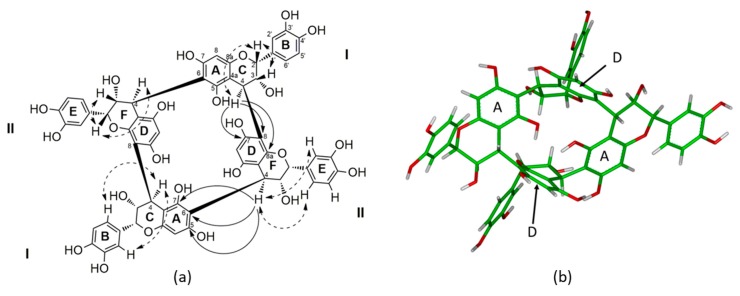
(**a**) Structure of the crown tetramer (**1**) with the key HMBC correlation (H→C, full arrow) and main ROESY correlation for the II units (dotted double arrow). Similar ROESY correlations were also observed for the I units. (**b**) 3D model of the crown procyadnidin tetramer (**1**) on the basis of NMR spectroscopic data and NMR restrained molecular modeling.

The ^1^H-NMR spectrum (Table 2) of this tetrameric structure appeared to be very similar to procyanidin dimer B2, with one drastic and specific difference. Indeed, the main difference was the lack of a pair of methylene protons between 2.5 and 3 ppm, which is characteristic of a proton on the carbon C-4 of the flavan-3-ol unit at the end of the polymeric chain (Figure 1). This lack of methylene proton proved the absence of flavan-3-ol as a terminal monomer, as observed during the acidic chemical depolymerization. Besides this unusual difference from regular oligomeric condensed tannin structures, the proton NMR spectra of **1** (Table 2) displayed two aromatic ABX system protons at δ 6.99 (2H, d, *J* = 1.8 Hz), 6.83 (2H, dd, *J* = 8.1, 1.8 Hz), and 6.71 (2H, d, *J* = 8.1 Hz) and at δ 6.42 (2H, d, *J* = 8.1 Hz), 6.20 (2H, d, *J* = 1.8 Hz), and 5.75 (2H, dd, J = 8.1, 1.8 Hz) respectively, with each accounting for two identical aromatic rings. Similarly, two distinct AMX-type resonances with each signal accounting for two protons were observed at δ 5.17 (2H, brs), 4.29 (2H, brd, *J* = 5.2 Hz), and 4.21 (2H, brd, *J* = 5.2 Hz) and at δ 4.56 (2H, brd, *J* = 2.5 Hz), 4.48 (2H, brd, J = 2.5 Hz), and 4.45 (2H, brs), which were assigned to H-2, H-3, and H-4 of the flavan-3-ol units. The appearance of two aromatic proton singlets at δ 6.09 and δ 6.12 indicated the occurrence of only two different sets of flavan-3-ol units linked through their C-6 or C-8 carbons, even if the compounds were tetramers. Indeed, according to the HMBC long-range correlations (Figure S4), each H-8 or H-6 proton exhibited a correlation with different C-4a and C-7. The assignation of H-8A was realized according to its specific HMBC long-range correlations with C-4aA, C-8aA, C-6A, and C-7A carbons, while H-6D was assigned according to its correlations with C-4aD, C-5A, C-6D, and C-7A carbons.

The interflavanyl linkages between these two sets of two identical flavan-3-ol units were confirmed by the strong HMBC (Figure 1 and Figure S4) correlations between H-4C (δ 4.21) and C-7D (δ 157.3), C-8D (δ 109.0), and C-8aD (δ 157.2), indicating that the interflavonoid linkage between Units I and II was 4→8, whereas the HMBC correlations between H-4F (δ 4.56) and C-5A (δ 155.5), C-6A (δ 106.3), and C-7A (δ 155.5) proved that the second interflavanyl linkage was 4→6 between Unit II and the second Unit I. These HMBC correlations also confirmed the lack of a terminal unit, since each C-4 carbon of each flavanyl unit were linked to the carbon C-8 or C-6 of another flavanyl unit.

Furthermore, the resonances of H-2C (δ 5.17), H-3C (δ 4.29), and H-4C (δ 4.21) for Unit I and H-2F (δ 4.45), H-3F (δ 4.48), and H-4F (δ 4.56) for Unit II exhibited small *J_2,3_* and *J_3,4_* coupling constants, indicating gauche dihedral angles together with the strong NOE correlations between H-2C (δ 5.17) and H-3C (δ 4.29) for Unit I and between H-2F (δ 4.45) and H-3F (δ 4.48) for Unit II on the ROESY spectrum, leading to the *cis*-orientation of these two pairs of protons, proving that each flavanyl unit is (−)-epicatechin (2,3-*cis*), which is consistent with the facts that only an (−)-epicatechin phloroglucinol adduct was obtained during chemical depolymerization. Indeed, such a strong NOE correlation is specific to and characteristic of (−)-epicatechin [17], while coupling constants of 8 to 10 Hz are specific to catechin units (2,3-*trans*) [18].

Finally, the orientation of each interflavanyl linkage at the C-4 position was assigned to be β orientated according to the positive Cotton effect between 220 and 240 nm observed on CD spectroscopy as previously reported in the literature [19]. Such β orientation was also supported by the chemical shift of C-2C (δ 78.5) and C-2F (δ 75.2) [17]. Indeed, in the case of an α-orientation, a much larger downfield C-2 chemical shift would be observed, like for the procyanidins B3 and B4 (δ 82 to 84) [17,20]. This β orientation also supports the correlations between H-4C (δ 4.21) and the proton H-2C (δ 517) as well as the aromatic protons of the B ring for Unit I and the correlations between H-4F (δ 4.56) and the proton H-2F (δ 4.45) as well as the aromatic protons of the E ring for Unit II [18]. Furthermore, the resonances of H-3C (δ 4.29) and H-4C (δ 4.21) for Unit I and H-3F (δ 4.48) and H-4F (δ 4.56) for Unit II exhibited a small *J_3,4_* coupling constant, which also supports the β-orientation of the interflavanyl linkages, since a *J_3,4_* coupling constant of about 8 Hz would have been expected for an α-orientation [20].

Thus, taking into consideration the molecular formula, the lack of terminal unit, the fact that the only linkage between the (−)-epicatechin moieties are β-orientated interflavanyl linkage C4-C8 and C4-C6, the structure of **1** has been determined to be a symmetric procyanidin with four (−)-epicatechin moieties linked together by a β-orientated B-type interflavanyl linkage to form the macrocyclic structure as reported in Figure 1. Since such a carbon skeleton has never been reported before for procyanidins in wine, or in the plant kingdom, we decided to name this new class of procyanidins “crown procyanidin tetramers.”

The stereochemistry of compound **1** was also supported by molecular modeling [21]. The relative configuration of **1** is in agreement with the 3D structure obtained by molecular modeling, taking into account all the NOE correlations observed in the ROESY spectra (Figure 1a and Figure S5). The 3D structure of **1** revealed an important cavity in the middle of the structure. Indeed the two aromatic D rings of the II units are parallel and distant of 5.54 Å, while on the other side the two aromatic A rings of Unit I are distant from 4.97 Å. Due to the high electronic density with the four aromatic rings, such cavity can be a putative site for chelation.

Following the identification and structural determination of this new procyanidin structure in red wine, Cabernet Sauvignon grapes harvested at maturity were delicately peeled to separate seeds, skins, and bunch stems prior to extraction. Each extract was then separately analyzed by UPLC-UV-Q-Tof in order to determine in which part of the berries (i.e., seed, skin, or bunch stem) the crown procyanidin tetramer **1** was located (Figure S6). Surprisingly, the crown procyanidin tetramer **1** appeared to be specifically localized in grape skin and totally absent in the seed or in the bunch stem, which is a drastic difference compared to non-cyclic procyanidin oligomers and polymers, which are present in seed, skin, and bunch stem. Such specificity raises questions regarding their biosynthesis and their role in plant growth and defense.

**Figure 2:**
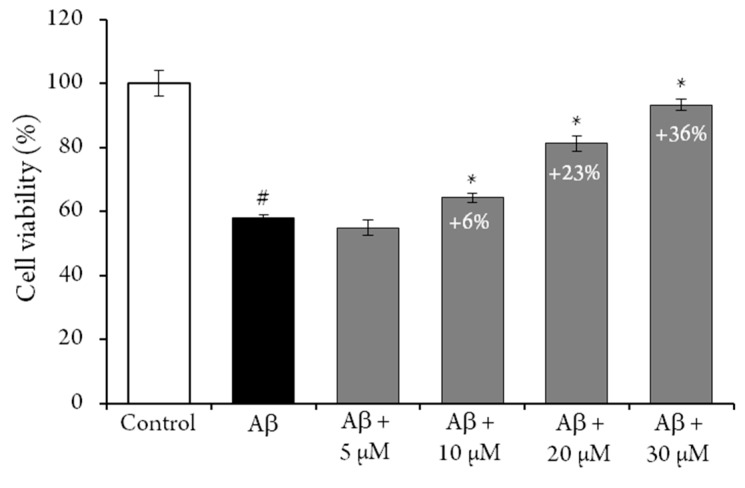
Cytotoxicity determination. PC12 cell viability was determined by the MTT assay. Cells were treated 24 h by the crown tetramer (**1**) in the presence or absence of 4 µM of Aβ25−35. Results are expressed as the mean SEM of four replicates (n = 4). # *p* < 0.05 Aβ25−35 versus control, * *p* < 0.05 crown tetramer (**1**) versus Aβ25−35.

The possible antiaging activities of compound **1** has also been evaluated, by measuring its capacity to protect rat neural pheochromocytoma-derived PC12 cells from amyloid-β peptide induced toxicity [21]. An MTT assay was used to determine cell viability, and results were expressed as the percentage of control cells. The crown tetramer (**1**) significantly protected PC12 cells from the cytotoxic effect of Aβ25−35 (Figure 2). After exposure to Aβ25−35 alone, the viability of PC12 cells was decreased to 42 ± 2%, and the addition of **1** increased this percentage in a dose-dependent manner. Indeed, the addition of 30 µM of **1** to the reaction media increased the viability of the PC12 cells (i.e., + 36%). However, when PC12 cells were exposed to the same concentration of **1** for 24 h, no difference was exhibited, compared to the control, as previously observed for flavan-3-ol and procyanidin dimer [22]. Similar results have been previously reported for resveratrol [23], while 50 µM cyanidin-3-*O*-glucoside has been reported to increase PC12 cells viability by only 10% [24]. These results indicate that **1** could be a candidate for novel neuroprotective strategies. Nevertheless, future studies evaluating the bioavailability and bioefficiency of this crown procyanidin should be conducted.

## 3. Materials and Methods

### 3.1. General Experimental Procedures

The optical rotation of the crown procyanidin tetramer was recorded on a Jasco P-2000 polarimeter using a sodium vapor light source and a wavelength set at 589 nm. The integration time (DIT) was 10 s. The concentration of the sample used was 2 mmol/L in pure water. The CD spectrum was recorded on a J-715 spectropolarimeter (Jasco France SAS, Lisses, France) in methanol (ca. 1 mg/mL) operating at 22 °C. Scan parameters: bandwidth, 1.0 nm; sensitivity, 100 mdeg; response, 1 scan speed, 50 nm/min; step resolution, 0.1 nm. The software used was Spectra Manager. The software used was Spectra Manager. Isolated crown procyanidin tetramer was analyzed by ^1^H-NMR and 2D-NMR spectroscopy (COSY, HSQC, HMBC, and ROESY) in methanol-*d_4_*. The spectra were recorded on AC 600 MHz and Avance III 600 MHz Bruker spectrometers (Wissembourg, France). Chemical shifts (δ) in ppm were referenced using the corresponding solvent signals: δ_H_ at 3.31 and δ_C_ at 49.00 for methanol-*d_4_*. Off-line FID processing was conducted with the Bruker TopSpin 3.2 software. Deionized water was purified with a Milli-Q water system (Millipore, Bedford, MA, USA). Methanol (HPLC grade), formic acid, and trifluoroacetic acid were purchased from Prolabo-VWR (Fontenay sous Bois, France). Water (Optimal^®^ LC/MS), methanol (Optimal^®^ LC/MS), and formic acid (Optimal^®^ LC/MS) were used for UPLC analysis coupled with a high resolution mass spectrometer was obtained from Fisher Scientific (Geel, Belgium). The LC-18 SPE cartridge column and the Toyopearl HW-40S were purchased from Sigma-Aldrich (Saint-Quentin Fallavier, France). Evaporations were conducted under reduced pressure at temperatures less than 40 °C.

### 3.2. Purification and Isolation

Fifteen milliliters of Cabernet Sauvignon red wine was evaporated to dryness and dissolved in 15 mL of water acidified with 0.1% formic acid. An SPE LC-18 cartridge column (10 g) was activated with 50 mL of methanol and washed with 100 mL of water to remove methanol. 15 mL of sample dissolved in water was applied to the column. Successive elutions were as follows: (1) 150 mL of methanol/water (5/95) to retrieve the tetramer, (2) 100 mL of methanol to wash the column, and (3) 100 mL of water to re-equilibrate the column. All solvents used were acidified with 0.1% formic acid. The fraction containing the tetramer was evaporated to dryness and re-dissolved in methanol. This solution was centrifuged twice for 3 min at 4500 R/min. The supernatant containing the tetramer was again evaporated and re-dissolved in 1 mL of water acidified with 0.1% formic acid. This solution was subsequently deposited on a gel column packed with Toyopearl HW-40S (d: 1 cm, h: 37.5 cm) and eluted with methanol with the flow rate of 0.8 mL/min. The fraction eluted between 16.5 and 22 h contained mainly the tetramer. This fraction was evaporated and re-dissolved in 300 µL of water acidified with 0.1% formic acid to be purified by semipreparative HPLC. The semi-preparative HPLC was performed on an Agilent 1260 Infinity system (Agilent, Les Ulis, France) containing a quaternary pump (1260 Infinity), a compartment thermostat column (1290 Infinity), a sample injector (1260 Standard Autosampler), and a diode array detector (1260 DAD VL+). A 500 µL external injector was added to the system. The semi-preparative column was a Prontosil column (250 × 8 mm, 5 µm, Metrohm France, Villebon-sur-Yvette, France). The solvents used were water/TFA (99.975/0.025, *v*/*v*) for Solvent A and methanol/TFA (99.975/0.025, *v*/*v*) for Solvent B. The flow rate was 2.5 mL/min. The gradient was as follows: 0% B remained for 5 min, 0–4% B for 30 min, 4–96% B for 2 min, and 96% B for 5 min. The column was re-equilibrated to initial conditions for 5 min after each injection. UV absorption spectra were recorded at 280 nm. The system was controlled using LC Open Lab software. Semi-preparative collected fractions containing the tetramer **1** exclusively were evaporated, frozen, and lyophilized to yield pure crown procyanidin tetramer as a white powder. Two liters of one-year-old Cabernet Sauvignon red wine (Bordeaux vintage 2013) was purified using this procedure, yielding 5.6 mg of the crown procyanidin tetramer.

*Crown procyanidin tetramer* (**1**): amorphous white powder; [α]${}_{D}^{20}$ 17.40 (c 0.002 H_2_O); ^1^H (methanol-*d*_4_, 600 MHz) NMR spectroscopic data, see Table 2; HRESIMS *m*/*z* 1153.2597 [M + H]^+^, calcd. for C_60_H_48_O_24_ 1153.2608.

### 3.3. Extraction from Grape

Cabernet Sauvignon grapes were harvested at maturity in September 2015 vineyards located in the Bordeaux wine-growing region in Southwestern France. Prior to the extraction, seeds, skins, and bunch stems from the frozen Cabernet Sauvignon grapes were delicately removed by hand and washed with distilled water to remove all the sugar from the rest of the pulp. Then, all the separated grapes were frozen and freeze-dried for 2 days and then ground to a powder with a mortar in order to be submitted to an extraction solvent. Exactly 0.5 g of each obtained powder was extracted using 5 mL of water/methanol (60:40, *v*/*v*) acidified with 0.1% of formic acid for 16 h under mechanical agitation. Then the solid part was separated from the solvent by centrifugation (3 min, 4500 rpm). The solid part was re-extracted a second time with the same solvent for 8 h under mechanical agitation and finally a third time in the same condition again for 16 h. All the liquid supernatant collected after centrifugation were mixed together, the solvent was evaporated, and the residue was dissolved in 2 mL of water, filtered (0.45 µm), and analyzed by UPLC-UV-Q-TOF. Each sample was extracted and analyzed in triplicate. The UPLC-MS system used was an Agilent 1290 Infinity (Agilent, Les Ulis, France) equipped with an ESI-Q-TOF-MS (Agilent 6530 Accurate Mass, Agilent, Les Ulis, France). Chromatographic separation was carried out on an Eclipse Plus C18 column (2.1 × 100 mm, 1.8 µm, Agilent, Les Ulis, France). The solvents used were as follows: water with 0.1% formic acid for Solvent A and methanol with 0.1% formic acid for Solvent B, with a flow rate of 0.3 mL/min. The gradient of Solvent B for was as follows: 6% for 0.5 min; 6–95% for 13.5 min; 95% for 4 min. The UPLC column was equilibrated for 3 min. The gradient of Solvent B for oligomers analysis was as follows: 4% for 10 min; 4–95% in 4 min; 95% for 4 min. The UPLC column was equilibrated for 3 min. ESI conditions were as follows: gas temperature and flow were 300 °C and 9 L/min, respectively; sheath gas temperature and flow were 350 °C and 11 L/min, respectively; capillary voltage was 4000 V. The collision energies used for MS^2^ were 15 and 30%. The fragmentor was always set at 200 V. The data obtained were treated by MassHunter Qualitative Analysis software (version B.05).

### 3.4. Molecular Modeling

Molecular modeling was conducted on a Silicon Graphics O_2_ workstation using the Molecular Simulation Incorporated software package (Accelrys, San Diego, CA, USA). Simulated annealing and energy minimization were done with Discover and NMR-Refine using the consistent-valence force field (cvff) model. Interproton distances were obtained from ROESY experiments. Quantitative determination of cross-peak intensities was calculated with Sparky. The NOE-distance constraints were estimated using a relation of the Braun model [25]. The simulation annealing calculation protocol involved 14 steps as described in a previous work [26]. The distance constraint was kept to 30 Kcal/mol/Å^2^.

### 3.5. Phloroglucinolysis

The purified tetramer fraction was dried under reduced pressure and dissolved in 500 µL of methanol. The depolymerization by phloroglucinolysis was conducted [16]. A phloroglucinol reagent solution containing 0.1 N HCl in methanol, 50 g/L phloroglucinol, and 10 g/L ascorbic acid was prepared. Two hundred microliters of the methanol solution containing the tetramer was added to 200 µL of the phloroglucinol reagent solution, and the reaction mixture was placed at 50 °C for 20 min. One milliliter of a 40 mM acetate sodium aqueous solution was added to stop the reaction. The depolymerized reaction mixture was analyzed by UPLC-DAD-ESI-Q-TOF system to identify the released monomeric unit.

### 3.6. Cell Culture and MTT Assay

PC12 cells derived from rat pheochromocytoma were cultured in a DMEM-Glutamax medium containing 100 units/mL penicillin, 100 µg/mL streptomycin, 15% fetal horse serum, and 2.5% fetal bovine serum and maintained at 37 °C in a humidified incubator with 5% CO_2_. In 200 µL of culture medium, PC12 cells were sub-cultured at a density of 30,000 cells per well, in 96-well culture plates. After 24 h, cells were incubated with 4 µM of Aß_25-35_ in the absence or presence of different concentrations of crown tetramer (5, 10, 20, and 30 µM), in a serum-free culture medium. After treatment (24 h), PC12 cells were incubated with 0.5 mg/mL of MTT for 3 h at 37 °C. The dark blue formazan crystals formed in intact cells were solubilized with DMSO for 0.5 h. The absorbance was measured at 595 nm with a microplate reader (Dynex, Carnegie, PA, USA). Results were expressed as the percentage of MTT reduction in relation to the absorbance of control cells at 100%. All data represent the average of three tests. Data are shown as means ± SEM.

## 4. Conclusions

A crown procyanidin tetramer (**1**) represents the first member of a new sub-family of condensed tannins, and crown procyanidins with other flavan-3-ol monomeric units would be expected in wine and in other beverages, as well as in plants extracts that exhibit non-cyclic condensed tannins, such as tea, cacao, and apples. Moreover from a plant physiological perspective the fact that the crown procyanidin tetramer appeared to be specifically localized in grape skin might indicate that these condensed tannins sub-family may play a specific role in plant growth and defense.

Moreover, the 3D structure of crown procyanidin tetramers exhibits an unusual cavity in the center of the molecule, which probably explains the higher polarity observed in liquid chromatography compared to regular oligomeric condensed tannins due to the formation of intramolecular hydrogen bonding and would offer unique binding possibilities. These binding possibilities would be even greater with crown procyanidin pentamers or higher oligomers that would exhibit larger cavities in the center of their structure.

## Figures and Tables

**Table 1:** Released product after chemical depolymerization of **1** detected by HPLC-Q-Tof.

Name	Measured *m*/*z* [M + H]^+^	Calculated *m*/*z* [M + H]^+^	Diff (ppm) ^1^	Chemical Formula
(−)-epicatechin phloroglucinol adduct	415.1023	415.1024	−0.24	C_31_H_18_O_9_
B-type dimer phloroglucinol adduct 1	703.1649	703.1657	−1.14	C_36_H_30_O_15_
B-type dimer phloroglucinol adduct 2	703.1653	703.1657	−0.57	C_36_H_30_O_15_
B-type trimer phloroglucinol adduct 1	991.2305	991.2291	1.41	C_51_H_42_O_21_
B-type trimer phloroglucinol adduct 2	991.2298	991.2291	0.71	C_51_H_42_O_21_

^1^ Mass difference between measured and calculated *m*/*z* express as ppm.

**Table 2:** ^1^H- and ^13^C-NMR assignments and HMBC correlations.

Position	δ_C_ Type	δ_H_ (Mult, *J* in Hz)	HMBC ^1^
2C	78.5	5.17, brs	3C, 4C, 8aA, 1’B, 2’B, 6’B
3C	73.0	4.29, brd, (5.2)	4C, 4aA, 8D, 1’B
4C	37.7	4.21, brd, (5.2)	8D, 8aD, 7D, 4aA, 8aA, 2C, 3C
4aA	104.9		
5A	155.5		
6A	106.3		
7A	155.5		
8A	96.1	6.12, s	4aA, 6A, 7A, 8aA
8aA	155.1		
1’B	131.9		
2’B	115.4	6.99, d, (1.8)	1’B, 3’B, 4’B, 6’B, 2C
3’B	145.4		
4’B	145.5		
5’B	115.5	6.71, d, (8.1)	1’B, 3’B, 4’B, 6’B
6’B	119.8	6.83, dd, (1.8, 8.1)	2’B, 4’B, 2C
2F	75.2	4.45, brs	3F, 4F, 1’E, 2’E, 6’E, 8aD
3F	67.9	4.48, brd, (2.5)	2F, 4F, 4aD, 1’E
4F	37.8	4.56, brd, (2.5)	5A, 6A, 7A, 4aD, 8aD, 2F, 3F
4aD	98.6		
5D	157.1		
6D	97.0	6.09, s	4aD, 5D, 7D, 8D
7D	157.3		
8D	109.0		
8aD	157.2		
1’E	131.3		
2’E	114.4	6.20, d, (1.8)	1’E, 3’E, 4’E, 6’E, 2F
3’E	144.8		
4’E	144.6		
5’E	115.5	6.42, d, (8.1)	1’E, 3’E, 4’E
6’E	119.7	5.75, d, (1.8, 8.1)	2F, 2’E, 4’E

^1^ HMBC correlations are from proton(s) stated to the indicated carbon.

## Supplementary Materials

Figure S1: (a) HRESIMS fragmentation spectra of the crown procyanidin tetramer; (b) Fragmentation pattern of the crown procyanidin tetramer. Figure S2: ^1^H-NMR spectrum (600 MHz, methanol-*d_4_*) of the crown procyanidin tetramer. Figure S3: HSQC spectrum (methanol-*d_4_*) of the crown procyanidin tetramer. Figure S4: HMBC spectrum (methanol-*d_4_*) of the crown procyanidin tetramer. Figure S5: ROESY spectrum (methanol-*d_4_*) of the crown procyanidin tetramer. Figure S6: UPLC-UV-QTOF chromatogram *m*/*z*: 1153.2597 A: bunch stem extract, B: grape seed, C: grape skin.
