# Supplementary material for: Crown Procyanidin Tetramer: A Procyanidin with an Unusual Cyclic Skeleton with a Potent Protective Effect against Amyloid-β-Induced Toxicity
Source: Molecules. 2019 May 18;24(10):1915. doi: 10.3390/molecules24101915 (PMC6572218; doi:10.3390/molecules24101915)
Supplement: Supplementary file 1 [file molecules-24-01915-s001.pdf]

molecules

MDPI

Supporting information

Crown Procyanidin Tetramer: A Procyanidin with Unusual Cyclic Skeleton with Potent Protective Effect against Amyloid-β-Induced Toxicity

Liming Zeng [1,2], Pere Pons-Mercadé [1,2], Tristan Richard [1,2], Stéphanie Krisa [1,2], Pierre-Louis Teissèdre [1,2] and Michael Jourdes [1,2,*]

[1] Univ. Bordeaux, ISVV, EA 4577, Œnologie, 210 Chemin de Leysotte, F-33140 Villenave d'Ornon, France; liming.zeng@changins.ch (L.Z.); pepome@gmail.com (P.P.-M.); tristan.richard@u-bordeaux.fr (T.R.); Stephanie.Krisa@u-bordeaux.fr (S.K.); pierre-louis.teissedre@u-bordeaux.fr (P.-L.T.)

[2] INRA, ISVV, USC 1366 INRA, IPB, 210 Chemin de Leysotte, F-33140 Villenave d'Ornon, France

* Correspondence: Michael.jourdes@u-bordeaux.fr

Table of Contents

Figure S1. (a) HRESIMS fragmentation spectra of the crown procyanidin tetramer; (b) Fragmentation pattern of the crown procyanidin tetramer

Figure S2. ^{1}H NMR spectrum (600 MHz, methanol-*d4*) of the crown procyanidin tetramer

Figure S3. HSQC spectrum (methanol-*d4*) of the crown procyanidin tetramer

Figure S4. HMBC spectrum (methanol-*d4*) of the crown procyanidin tetramer

Figure S5. ROESY spectrum (methanol-*d4*) of the crown procyanidin tetramer

Figure S6. UPLC-UV-QTOF chromatogram m/z: 1153.2597 **A**: bunch stem extract, **B**: grape seed, **C**: grape skin

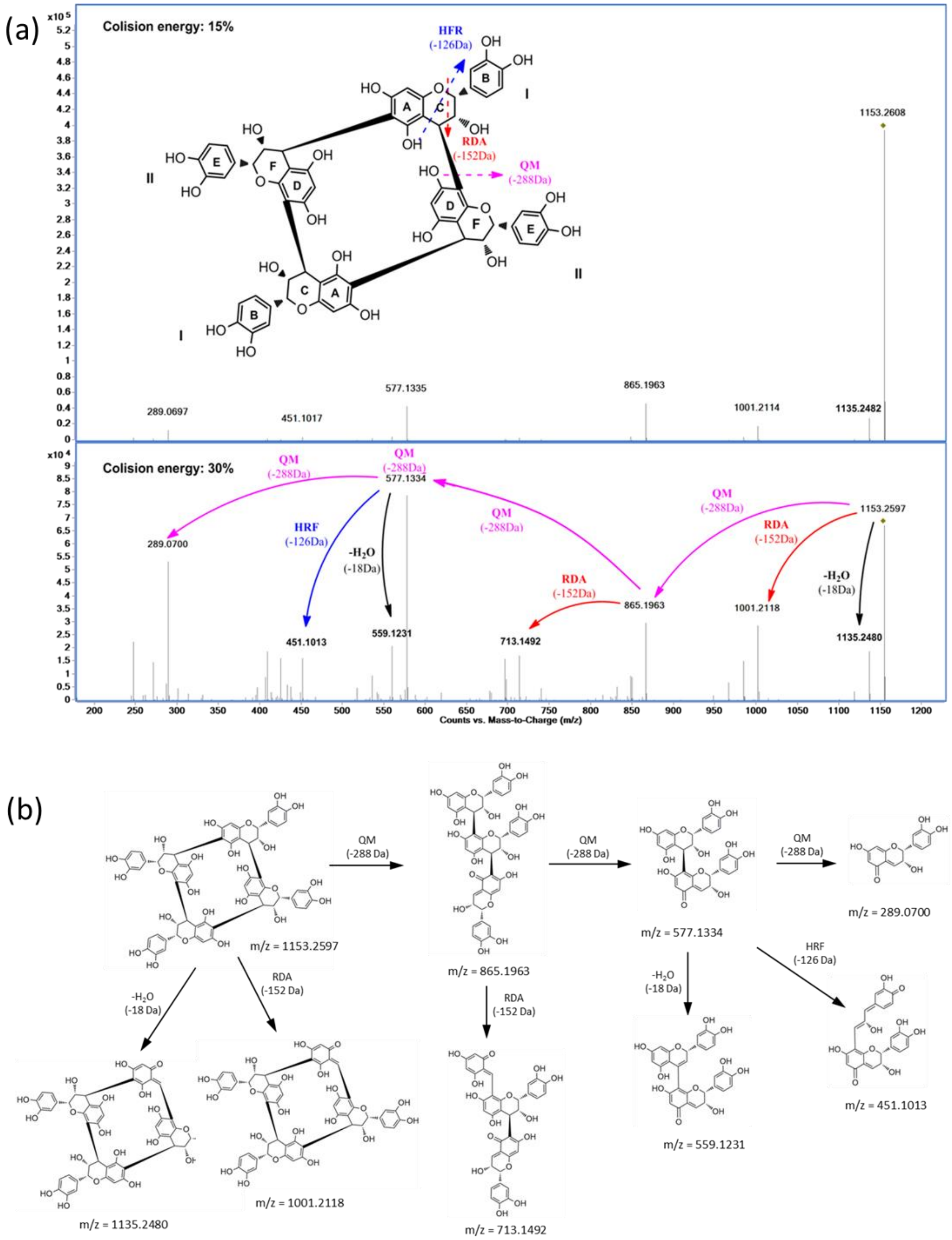

Figure S1. (a) HRESIMS fragmentation spectra of the crown procyanidin tetramer; (b) Fragmentation pattern of the crown procyanidin tetramer

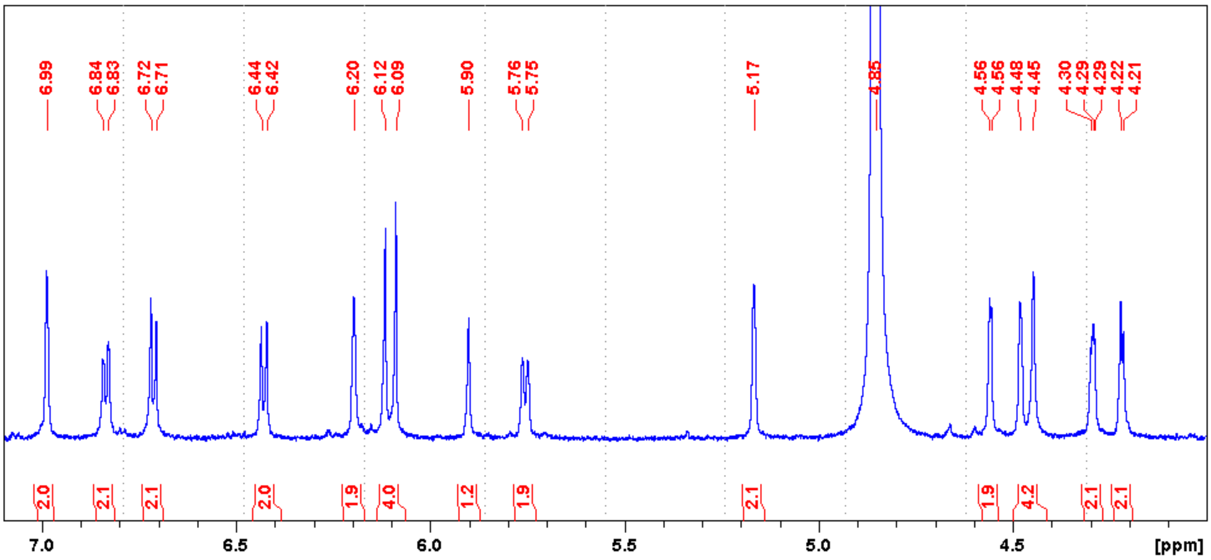

Figure S2. ^{1}H NMR spectrum (600 MHz, methanol-*d4*) of the crown procyanidin tetramer

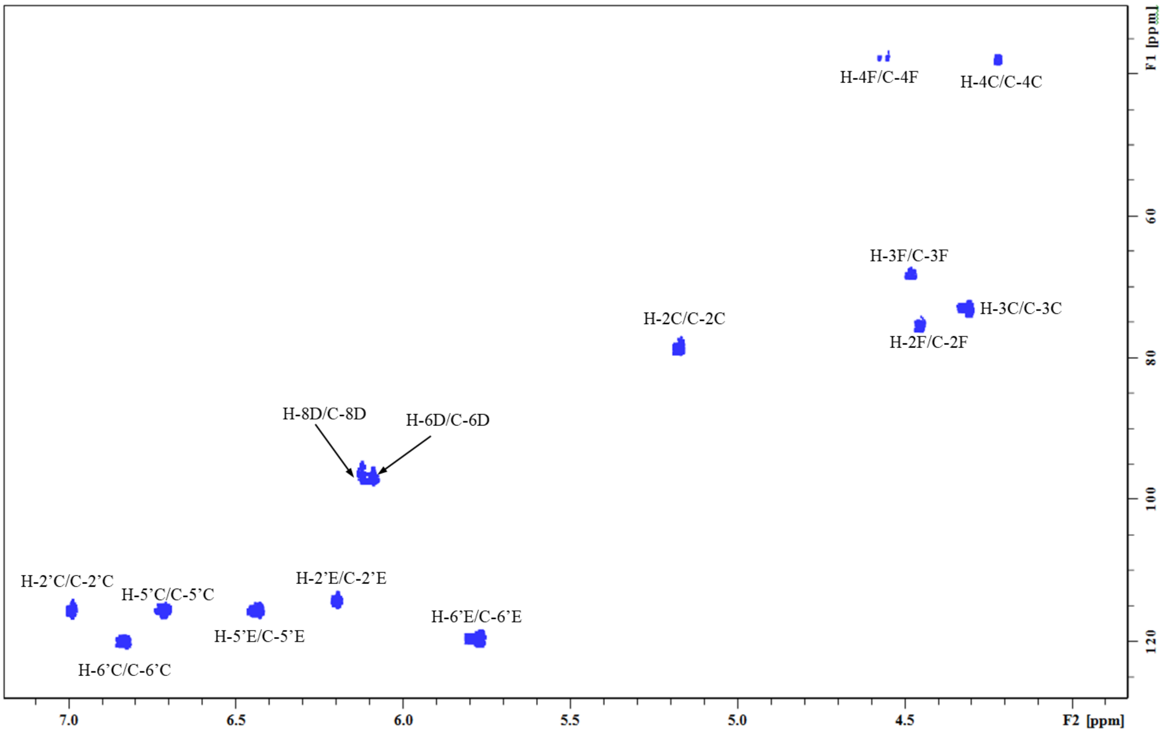

Figure S3. HSQC spectrum (methanol-*d4*) of the crown procyanidin tetramer

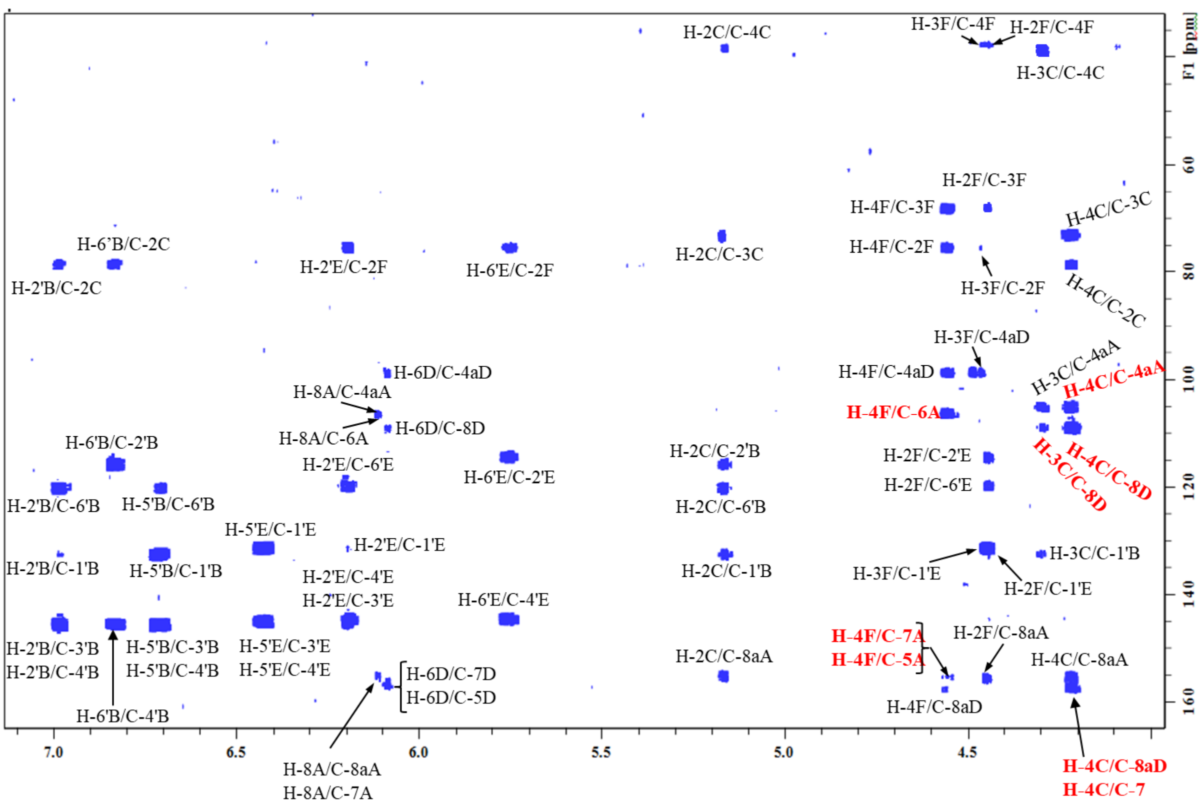

Figure S4. HMBC spectrum (methanol-*d4*) of the crown procyanidin tetramer

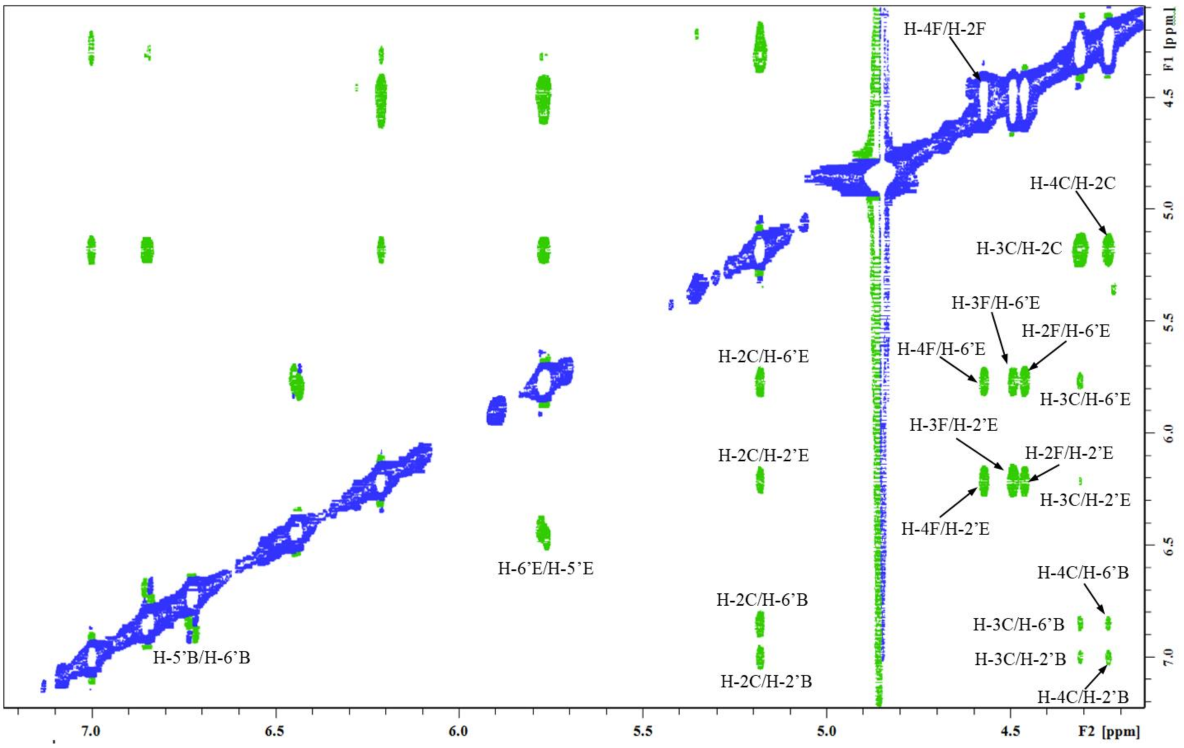

Figure S5. ROESY spectrum (methanol-*d4*) of the crown procyanidin tetramer

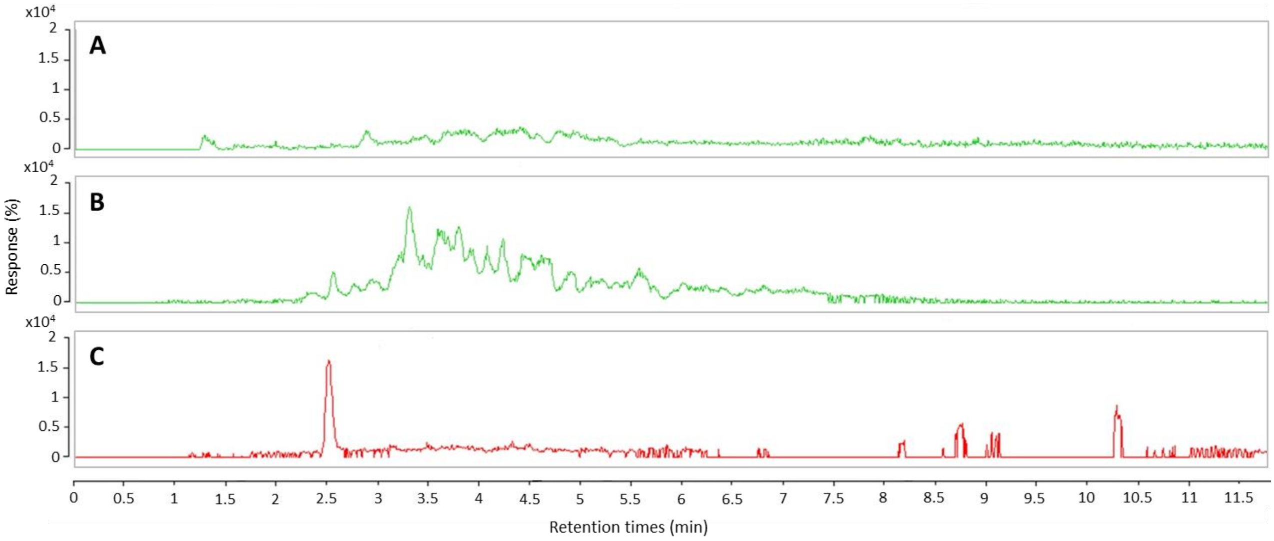

Figure S6. UPLC-UV-QTOF chromatogram m/z: 1153.2597, **A**: bunch stem extract, **B**: grape seed, **C**: grape skin

Sample Availability: Samples of the compounds ...... are available from the authors.

© 2019 by the authors. Licensee MDPI, Basel, Switzerland. This article is an open access article distributed under the terms and conditions of the Creative Commons Attribution (CC BY) license (http://creativecommons.org/licenses/by/4.0/).
